# Traumatic Brain Injuries during Development: Implications for Alcohol Abuse

**DOI:** 10.3389/fnbeh.2017.00135

**Published:** 2017-07-20

**Authors:** Zachary M. Weil, Kate Karelina

**Affiliations:** Behavioral Neuroendocrinology Group, Department of Neuroscience, Center for Brain and Spinal Cord Repair, Ohio State University Wexner Medical Center Columbus, OH, United States

**Keywords:** alcohol, traumatic brain injury, dopamine, inflammation, adolescent

## Abstract

Traumatic brain injuries are strongly related to alcohol intoxication as by some estimates half or more of all brain injuries involve at least one intoxicated individual. Additionally, there is mounting evidence that traumatic brain injuries can themselves serve as independent risk factors for the development of alcohol use disorders, particularly when injury occurs during juvenile or adolescent development. Here, we will review the epidemiological and experimental evidence for this phenomenon and discuss potential psychosocial mediators including attenuation of negative affect and impaired decision making as well as neurochemical mediators including disruption in the glutamatergic, GABAergic, and dopaminergic signaling pathways and increases in inflammation.

## Introduction

Traumatic brain injuries (TBI) have received tremendous scientific and public attention in recent years. This attention is commensurate with the enormous societal and economic costs associated with a condition that affects millions of Americans and because of underreporting is likely even larger than current estimates. One important insight that has become clear is that TBIs are more than discrete events but are the start of a lifelong process comprising recovery, adaptation, and vulnerability to a large variety of other disease states (Masel and DeWitt, 2010; Corrigan and Hammond, 2013). In particular, children and adolescents who suffer TBI are less likely to finish school and maintain employment and suffer a greater risk of neurological and psychiatric disorders (among many others; Corrigan et al., 2013).

Alcohol is a prominent component of TBI. A recent assessment of the U.S. national trauma registry revealed alcohol use to be present in as many as 50% of all TBI-related emergency department visits (Chen et al., 2012). Not surprisingly alcohol use in general and binge drinking in particular are powerful risk factors for TBI (Savola et al., 2005; Vaaramo et al., 2014) and contribute substantially to mortality of TBI patients (see reference Opreanu et al., 2010 for a review). Critically, the use of alcohol in patients recovering from TBI is highly deleterious and there is significant evidence that patients that drink after TBI have poorer cognitive, neuropsychiatric and occupational outcomes than those that do not (Corrigan, 1995; Weil et al., 2016a; Unsworth and Mathias, 2017). Drinking after TBI is associated with poor long term outcomes in a variety of domains (Corrigan, 1995). Similar results have been reported in experimental TBI, for example we recently reported that administration of binge-like levels of alcohol in adulthood produces significant functional and neuropathological impairments in mice that had experienced TBI as juveniles (Karelina et al., 2017). Given that both past TBI and drinking are risk factors for future TBI *and* repeated TBI tend to produce much more severe damage (Guskiewicz et al., 2000; Giza et al., 2013; McCrory et al., 2013) reducing drinking behavior in this patient population will serve to both improve outcomes and reduce the possibility of devastating future injuries.

The strong epidemiological association between brain injuries and pre-injury drinking mean that the TBI population is composed disproportionately of heavy drinkers. However, there is emerging clinical and experimental evidence that TBI may serve as an independent risk factor for the development of alcohol use disorders (AUDs; Weil et al., 2016a; Merkel et al., 2017a). This is particularly apparent among patients that suffer TBIs during childhood or adolescence. The TBI-induced increase in alcohol abuse among patients that suffer injuries during development likely reflects both a greater vulnerability of the developing nervous system to disruption by injury and that children are less likely to already be problem drinkers at the time of their injuries, and thus it is easier to detect independent contributions of TBI to the development of AUDs (Weil et al., 2016a).

## Drinking in TBI patients

The relationship between TBI and alcohol abuse is well-known but had been considered to be unidirectional, i.e., drinking was a risk factor for head injuries. The possibility that the opposite was also true, i.e., that brain injuries could under specific conditions increases drinking behavior, was obscured by several factors. First, problem drinking (particularly binge-drinking) is a key predictor and proximate cause of TBI (Corrigan and Mysiw, 2012). Therefore, the TBI population is made up disproportionately of heavy drinkers making it difficult to detect effects of TBI on later alcohol-related outcomes (Corrigan, 1995). Second, patients with the most severe injuries often reduce drinking during the first months after injury. This seems to occur because of a combination of factors but includes lack of access to alcohol because of physical disability and hospitalization (and inpatient rehabilitation; Bombardier et al., 2003). Third, most studies have examined individuals injured as adults but that represents both a relatively small proportion of the total population and includes individuals that have either already begun drinking or are past the age at which new AUD tend to emerge (Grant and Dawson, 1997). Finally, patients that begin (or resume) drinking after injury are more likely to be lost to follow up in longitudinal studies and thus the numbers of these individuals might be underestimated (Corrigan et al., 1997). Indeed, studies of post-TBI drinking in adults have most often reported that there is an initial decrease in alcohol intake followed by some patients gradually returning to problem drinking and others becoming abstainers (Kreutzer et al., 1996; Ponsford et al., 2007). Studies have produced conflicting reports as to whether adult injuries increase the rates of, or vulnerability to, AUDs (Bjork and Grant, 2009).

In contrast, early life injury has been consistently and repeatedly associated with the later development of AUDs (Weil et al., 2016a; Merkel et al., 2017a). For instance, high school students that suffered a TBI were more than twice as likely to meet the diagnostic criteria for AUDs after injury (Ilie et al., 2015). Further the earlier that injuries occur the stronger the association with substance abuse. Children injured before age five were more than 3.6 times as likely to exhibit substance abuse as teenagers than were uninjured children (McKinlay et al., 2014). Patients in an inpatient rehabilitation setting for TBI were more than twice as likely to meet the diagnostic criteria for substance abuse if they had experienced a previous injury before the age of 16 (Corrigan et al., 2013). In addition, among inmates in the South Carolina penitentiary system, age of first brain injury was associated with both severity and earlier age of onset of substance abuse (Fishbein et al., 2016).

A similar age of injury-related discrepancy in drinking after TBI has also been reported in animals. We recently reported that juvenile TBI, but not adult injury, significantly increased alcohol self-administration in a two-bottle choice paradigm in mice (Weil et al., 2016b). Other investigations of drinking behavior in animals injured as adults have produced conflicting results with some studies reporting increases and others reporting decreases (Lowing et al., 2014; Lim et al., 2015; Mayeux et al., 2015). Taken together, it seems that injuries that occur early in life are more likely to produce AUD, however, the specific mechanisms that link TBI to vulnerability to problem drinking remain unspecified.

## Potential mechanisms of increased drinking after TBI

**Figure 1:**
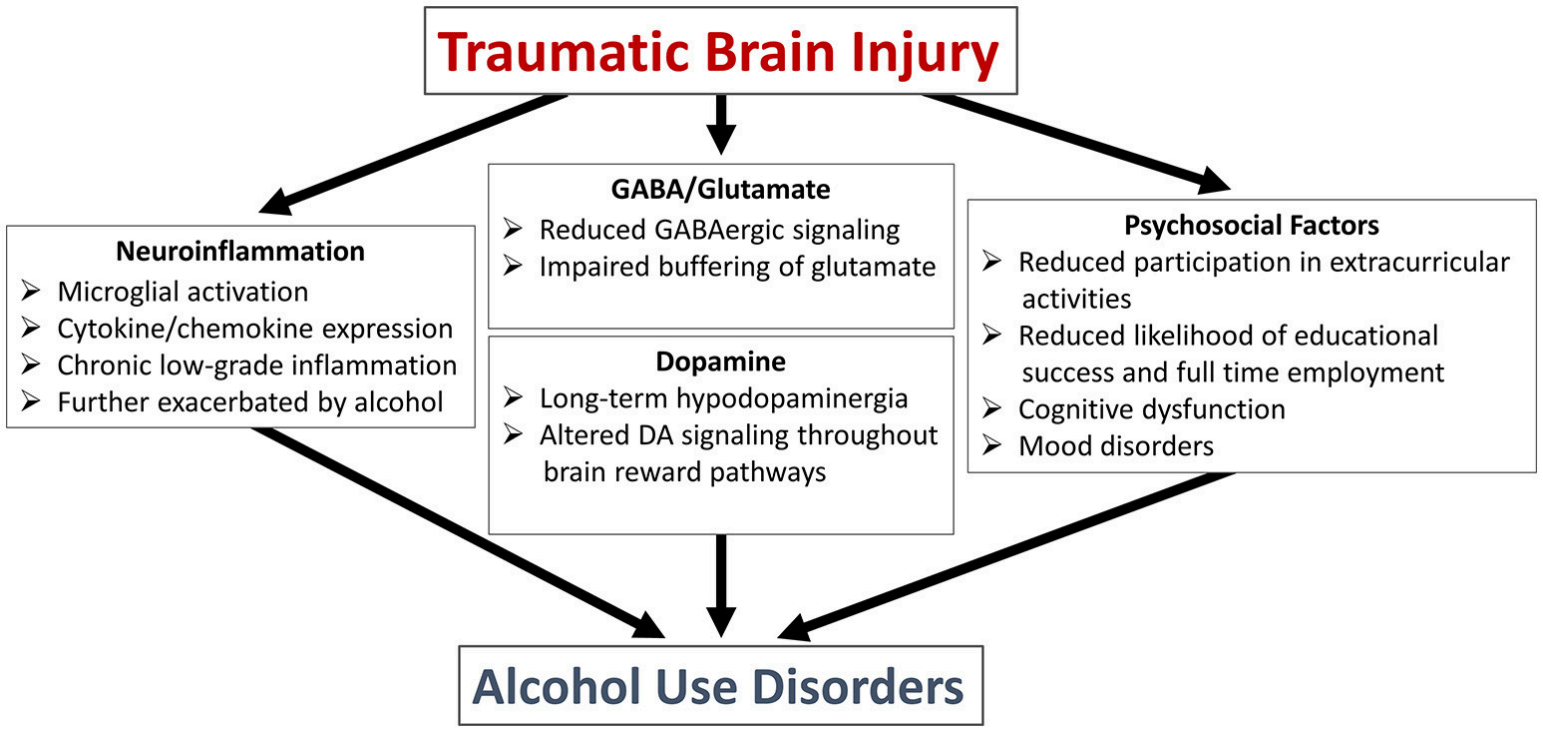
Potential mechanisms linking traumatic brain injury to alcohol use disorders.

In the next section we will discuss two very general classes of potential mediators that underlie the increased drinking behavior observed in pediatric brain injury patients (summarized in Figure 1). This is not intended to be an exhaustive discussion but rather to highlight some of the active areas of research.

## Psychosocial factors

TBI interacts with or exacerbates many of the traditional risk factors for AUD and may limit some of the moderating factors that serve to reduce AUD risk. Although, the individual contributions of these risk factors are likely relatively minor, when taken together they contribute to the increased likelihood of AUD in these patients. For example, many head injury patients have a family history of AUD, which results in both increased modeling of drinking behavior and genetic risk (Laucht et al., 2007). In addition, head injuries in pediatric populations are strongly, but often indirectly, associated with alcohol intoxication. In children and adolescents, vehicular accidents and physical violence are responsible for a substantial subset of injuries (though the exact percentages shift across age; Keenan and Bratton, 2006; Narang and Clarke, 2014) and extremely large percentages of head injuries related to assaults or incidences of physical abuse are associated with intoxication of either the injured child or more often the assailant, who is often a parent or other close relative (Kraus et al., 1989; Dube et al., 2001). A similar relationship exists for motor vehicle accidents where at least one participant is likely to be intoxicated (Stoduto et al., 1993). Further, male children are more likely to experience abuse-associated head trauma and are also at greater risk of later TBI and adolescent AUD (Costello et al., 1999; Narang and Clarke, 2014). Thus, the pediatric TBI population consists disproportionately of individuals from a family with alcohol use problems and/or a history of abuse, which are both critical risk factors for the development of AUDs (Dube et al., 2001; Barnow et al., 2002).

TBI also reduces many of the negative predictors of substance abuse. For instance, participation in extracurricular activities, forming stable romantic relationships, educational success, and full time employment are associated with reduced risk of alcohol abuse, yet all are less likely in brain injury survivors (Stewart-Scott and Douglas, 1998; Corrigan et al., 2013). Further, more severe TBI is often associated with prolonged absence from school and long-term disability, which often results in alienation from peer groups and increased alcohol use (Glang et al., 1997; Maggs et al., 2008).

The decision to drink has been proposed to reflect a balance between potential negative consequences (hangover, relationship issues, health consequences etc.) and perceived gains (reducing social inhibitions, negative mood states etc.) (Goldman, 1994). TBI can influence both sides of that equation by both reducing the ability to perceive negative consequences and increasing the perception of gains to be derived from drinking. For instance, that framework, termed the incentive motivation theory (Goldman, 1994), would predict that individuals less able to foresee the future consequences of their actions would be more likely to elect to drink. Indeed, TBI survivors show deficits in delay discounting and other cognitive tasks that require evaluation of delayed consequences (Bechara et al., 1994; Kolitz et al., 2003; Graham and Cardon, 2008). Finally, TBI results in more general executive dysfunction that can manifest itself as impulsivity and reduced inhibition, which can serve as predictors of AUD (Laucht et al., 2007; Iacono et al., 2008).

The other component of the incentive motivation theory is that individuals perceive a benefit to drinking. This perceived benefit could be in the form of increasing mood state, or reducing negative emotions or pain, and thus serves as a form of self-medication (King et al., 2004; Bolton et al., 2009). TBI survivors suffer from psychiatric sequelae including anxiety, depression and in a large subset of cases, post-traumatic stress disorder (PTSD; Breslau et al., 1991, 1997; Jorge et al., 2004). TBI often damages corticolimbic structures that regulate mood states, and endocrine and autonomic physiology as well as inducing long-term inflammatory responses, which can all increase the symptoms of affective disorders (Juengst et al., 2015). Even beyond direct damage to the nervous system, patients that acquire long-term cognitive or physical disabilities after brain injury often undergo a significant and prolonged period of adjustment to living with a disability (Smedema and Ebener, 2010). Thus, the very real possibility exists that brain injured patients are drinking to reduce the negative emotional states that are promoted by brain injury (Beresford et al., 2005).

## Neuroinflammatory signaling

The psychosocial and genetic risk factors associated with pediatric TBI also occur in the context of damage to the developing nervous system and likely disruptions of normal brain development. TBI is a complex pathophysiological process that can involve neuronal death, axon disconnection and degeneration, metabolic dysfunction, and aberrant neuroplasticity among other processes depending on the exact type and severity of the injury, comorbidities and age of the patient (Werner and Engelhard, 2007). However, one feature nearly universal to TBI is inflammation (Kelley et al., 2007; Ziebell and Morganti-Kossmann, 2010; Johnson et al., 2013). TBI both directly activates immune cells in the brain and primes cells such that future inflammatory stimuli produce exaggerated inflammatory responses (Fenn et al., 2014) and this is particularly true when inflammatory events occur early in life (Bilbo and Schwarz, 2009). The enhanced basal and stimulus-evoked immune responsiveness of the injured nervous system is important because there is a bidirectional relationship between neuroinflammation and alcohol intake (Kelley and Dantzer, 2011).

Alcohol produces a central inflammatory response characterized by activation of microglia and induction of inflammatory signaling and cytokines (Valles et al., 2004; Crews et al., 2011). Indeed the brains of long-term alcoholics exhibit evidence of prolonged low-grade inflammatory responses that may contribute to cognitive decline (He and Crews, 2008; Leclercq et al., 2014; Yen et al., 2017). The specific mechanism through which alcohol induces inflammatory responses is not fully understood but likely includes activation of the danger signal detecting molecules toll-like receptors (TLR; Alfonso-Loeches et al., 2010; Pascual et al., 2011). Critically, inflammation and components of inflammatory signaling drive drinking behavior (Crews et al., 2011). Mice treated with the bacterial cell wall component lipopolysaccharide (a molecule that induces a potent inflammatory response) self-administer significantly more alcohol (Blednov et al., 2011). Further, treatment with minocycline, a semisynthetic antibiotic with potent central anti-inflammatory activity produces a prolonged reduction in spontaneous alcohol self-administration (Agrawal et al., 2011). Mice lacking various components of inflammatory signaling cascades also drink less under basal conditions (Blednov et al., 2012).

Thus, TBI produces both acute inflammatory responses *and* primes immune cells like microglia to exhibit exaggerated inflammatory responses to other stimuli later in life. In this manner, TBI can establish a vicious cycle wherein inflammatory responses promote drinking behavior and subsequent drinking further exacerbates inflammatory responses (Mayeux et al., 2015). Critically, the alteration in inflammatory responses from alcohol occur in a brain already impacted by TBI and thus in addition to the deleterious consequences of heavy drinking that occur in otherwise healthy individuals, TBI patients face the potential of enhanced and chronic neuroinflammatory responses.

## Neurochemical abnormalities

Dysfunction in neuronal signaling after TBI, during development, can be roughly categorized into several etiologies. First, there is some (although often minimal) frank loss of neurons and associated axonal degeneration that directly disconnects or otherwise impairs neuronal connections. Additionally, as many critical neurodevelopmental events are occurring during these developmental epochs, TBI can result in disruption in the establishment of, or homeostasis in, neurochemical systems.

For instance, the ascending dopaminergic system undergoes significant functional and anatomical plasticity during late childhood and early adolescence (Philpot et al., 2009). This is characterized by changing tonic and stimulus evoked firing, alterations in synthetic machinery, transporter expression, and receptor distribution (Wahlstrom et al., 2010; McCutcheon et al., 2012). Importantly, this period of rapid neurodevelopment coincides temporally with vulnerability to substance abuse (Grant and Dawson, 1998). Early experience with drugs of abuse is a key risk factor for the development of substance abuse disorders and has been shown experimentally to alter the long-term function of the dopamine system (Guerri and Pascual, 2010).

Similarly, dysfunction in dopaminergic signaling is both a common consequence of TBI and a risk factor for the development of AUD (Martinez et al., 2005). There is a large body of experimental animal work indicating that dopamine physiology is significantly altered by TBI, with most studies reporting an acute hyperdopaminergia that resolves into a long-term hypodopaminergic state (Yan et al., 2001, 2002; Wagner et al., 2005a,b, 2009; Hutson et al., 2011).

Although, there is little direct evidence of dopaminergic dysfunction in human TBI patients (Donnemiller et al., 2000), drugs that enhance dopaminergic signaling (either by increasing synaptic dopamine or directly agonizing dopamine receptors) are relatively effective and part of the standard of care for reducing the cognitive deficits experienced by TBI patients (Neurobehavioral Guidelines Working et al., 2006; Bales et al., 2009; Huang et al., 2016). The utility of these drugs does not necessarily indicate that the dopamine system is hypofunctional in human patients (i.e., more dopamine may be helpful to improve cognitive outcomes in patients because of other neurological deficits) but the preponderance of evidence from clinical and experimental sources suggest some level of long term dysfunction in this system (Bales et al., 2009).

Critically, the alterations in dopamine signaling appear to occur beyond what would be expected from frank axonal degeneration or neuronal death (although damage to the ventral tegmental area and striatal targets have been reported following TBI; Dunn-Meynell and Levin, 1997; Ding et al., 2001; Hutson et al., 2011). Rather the alterations in dopamine signaling likely include alterations in network regulation and ongoing inflammation (Merkel et al., 2017a,b). Inflammatory signaling also serves to impair dopaminergic signaling and likely plays a role in the vulnerability to substance abuse in the brain-injured population (Felger and Miller, 2012). For instance, tyrosine hydroxylase production of L-Dopa is the rate-limiting step in dopamine biosynthesis. This enzymatic reaction requires the cofactor, tetrahydrobiopterin (BH4), which is also required for the synthesis of nitric oxide by nitric oxide synthases which are strongly upregulated by inflammatory signals meaning that BH4 can be shunted away from tyrosine hydroxylase when the brain is inflamed (Cunnington and Channon, 2010; Ono et al., 2010; Felger and Miller, 2012).

In contrast, surprisingly little is known about long-term adjustments in the glutamatergic and GABAergic systems after TBI. Acutely, TBI is associated with large, uncontrolled glutamate release that is a key factor in the damage associated with trauma (Katayama et al., 1990; Bullock et al., 1998). Further GABAergic neurons may be disproportionately likely to die and are overall less effective at balancing excitation. Finally, there is often persistent dysfunction in glial cells that typically buffer extracellular glutamate concentrations by expressing transporter proteins. Together, these lead to a net increase in excitatory signaling (Cantu et al., 2015) and the dysregulation in excitatory- inhibitory balance is very often associated with the development of post-traumatic epilepsy, particularly after pediatric injury (Ates et al., 2006; Pavlov et al., 2011). Over the long term there are compensatory changes that seem to buffer excess excitation but as a consequence of limiting excitatory neurotransmission may limit cognitive recovery (De Beaumont et al., 2012). This is evidenced by the consistent finding that TBI impairs the expression of long-term potentiation (Giza and Prins, 2006; Schwarzbach et al., 2006; Dorsett et al., 2017).

Like TBI, AUD are associated with disruption in the balance in excitatory-inhibitory balance and dysregulation of both glutamatergic and GABAergic signaling (Koob and Volkow, 2016). Alcohol both directly modulates activity of glutamate and GABA receptors, and can induce compensatory adjustments in these systems that perpetuate problem drinking (Chandler, 2003; Fitzgerald et al., 2012). The dynamic role of GABA:glutamate dysregulation and the interaction with normal development of this system in the evolution of drinking after TBI remains unspecified but deserves further attention.

## Concluding remarks

Alcohol use among adolescents is exceptionally common in western societies. Many individuals can drink heavily during this developmental period without developing AUD. However, patients with a history of TBI are much more likely to develop AUD. This is a major and critical public health problem because drinking after TBI can increase the risk of post-traumatic seizures, impair the efficacy of rehabilitation programs and increase the likelihood of subsequent TBI. The specific mechanisms that link TBI to AUD remain unspecified but it seems highly likely that it involves the coincidence of key psychosocial and neurochemical risk factors with important periods of neurological development. Targeting AUD in this population has the potential to significantly improve long-term outcomes.

## Author contributions

All authors listed have made a substantial, direct and intellectual contribution to the work, and approved it for publication.

### Conflict of interest statement

The authors declare that the research was conducted in the absence of any commercial or financial relationships that could be construed as a potential conflict of interest.
